# Supplementary material for: Lung Mycobiota α-Diversity Is Linked to Severity in Critically Ill Patients with Acute Exacerbation of Chronic Obstructive Pulmonary Disease
Source: Microbiol Spectr. 2023 Mar 28;11(2):e05062-22. doi: 10.1128/spectrum.05062-22 (PMC10100765; doi:10.1128/spectrum.05062-22)
Supplement: Supplemental file 1 — Supplemental material. Download spectrum.05062-22-s0001.pdf, PDF file, 1.4 MB [file spectrum.05062-22-s0001.pdf]

Supplemental Figure 1. Non metric Bray-curtis analysis of β-diversity of the ITS2 sequencing run. Mock: mock community. TN: negative control from extraction step. TN PCR: negative control from amplification PCR step.

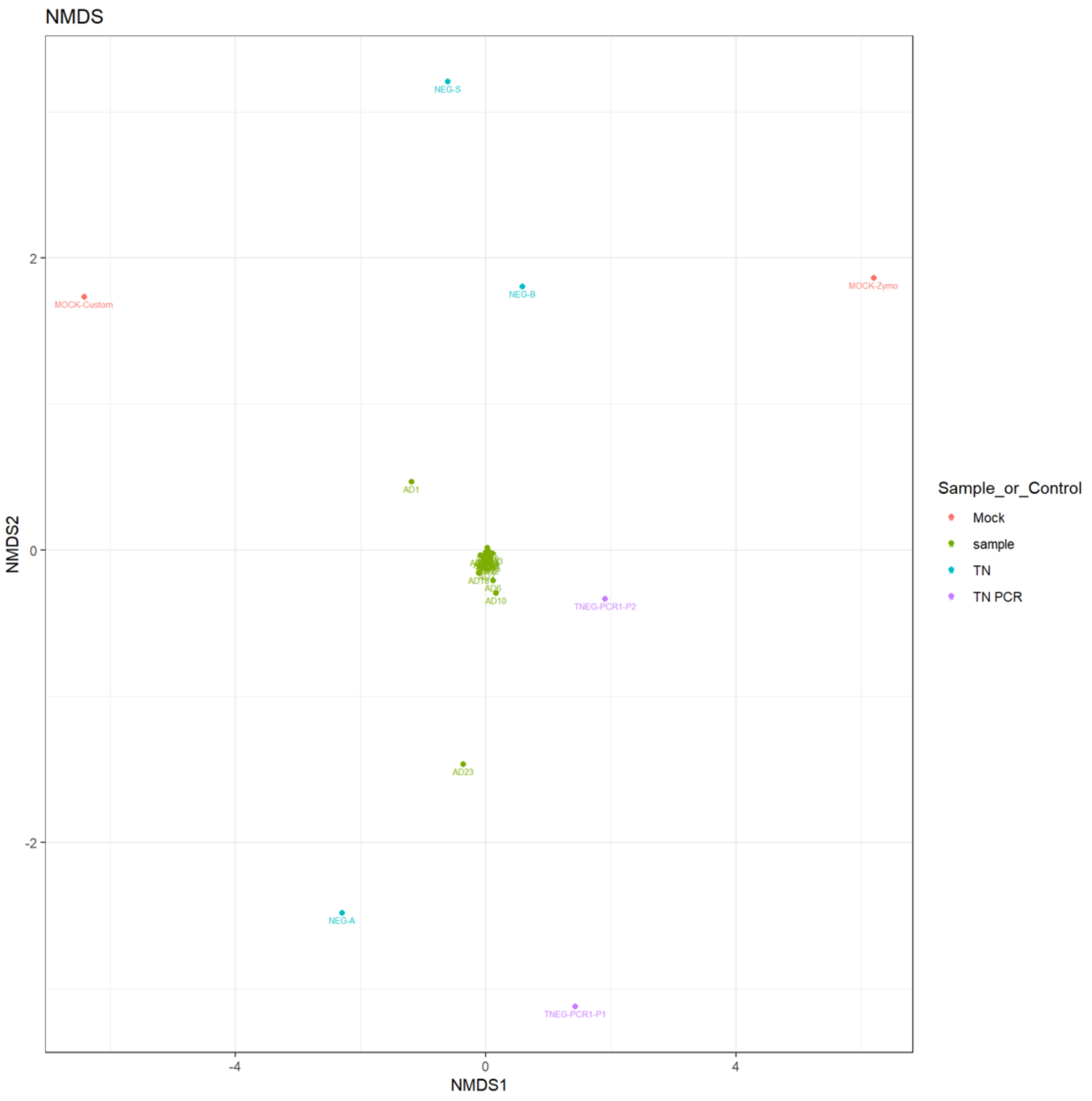

Supplemental Figure 2. Non metric Bray-curtis analysis of β-diversity of the V3-V4 sequencing run. Mock: mock community. TN: negative control from extraction step. TN PCR: negative control from amplification PCR step. AD5, AD9, AD22 and AD32 were excluded from further analyses.

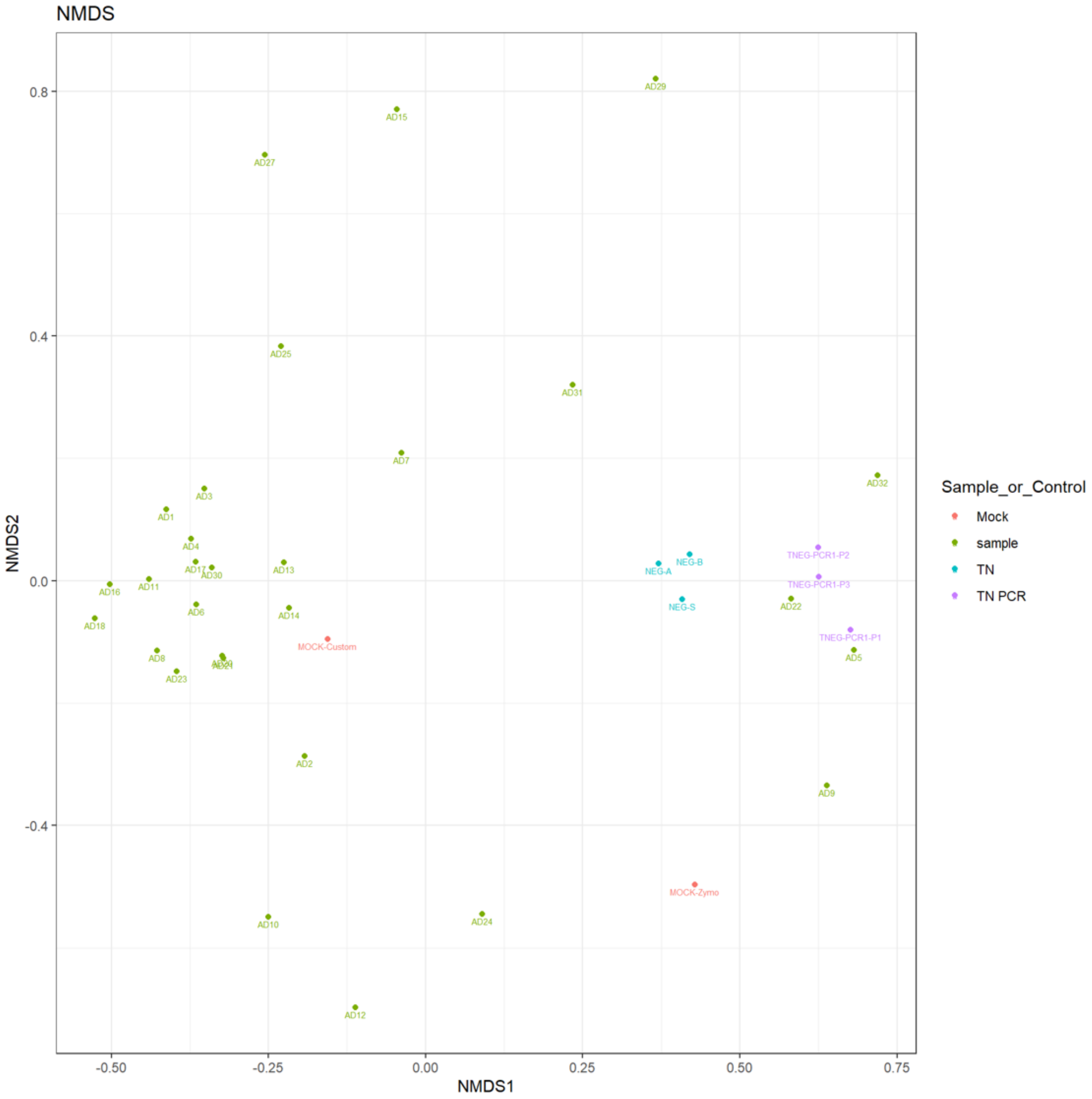

Supplemental Table 1. Number of ITS 2 reads before and after filtration.

sample_name .phylo.	Initial number of reads	Filtered reads	proportion	Number of reads after filtration
AD1	25992	25652	98.691905	340
AD10	16998	15274	89.857630	1724
AD11	34967	34816	99.568164	151
AD12	22339	22231	99.516541	108
AD13	6949	6935	99.798532	14
AD14	18341	18216	99.318467	125
AD15	47910	47805	99.780839	105
AD16	24366	23958	98.325536	408
AD17	48520	48270	99.484749	250
AD18	65413	64680	98.879428	733
AD19	57233	56528	98.768193	705
AD2	44127	41598	94.268815	2529
AD20	34725	34645	99.769618	80
AD21	28085	28051	99.878939	34
AD22	13456	12876	95.689655	580
AD23	18134	3524	19.433109	14610
AD24	37321	37094	99.391763	227
AD25	50444	50059	99.236777	385
AD26	61296	60833	99.244648	463
AD27	46930	45715	97.411038	1215
AD28	37894	37222	98.226632	672
AD29	45112	44856	99.432524	256
AD3	41115	40638	98.839839	477
AD30	36280	35008	96.493936	1272
AD31	46279	46200	99.829296	79
AD4	26507	26369	99.479383	138
AD5	33582	32984	98.219284	598

sample_name .phylo.	Initial number of reads	Filtered reads	proportion	Number of reads after filtration
AD6	39509	37631	95.246653	1878
AD7	44785	44141	98.562018	644
AD8	34596	34531	99.812117	65
MOCK-Custom	8327	0	0.000000	8327
MOCK-Zymo	27344	455	1.663985	26889
NEG-A	356	117	32.865169	239
NEG-B	198	169	85.353535	29
NEG-S	1738	314	18.066743	1424
TNEG-PCR1-P1	9	9	100.000000	0
TNEG-PCR1-P2	66	23	34.848485	43

Supplemental Figure 3. Rarefaction curves for ITS2 sequencing

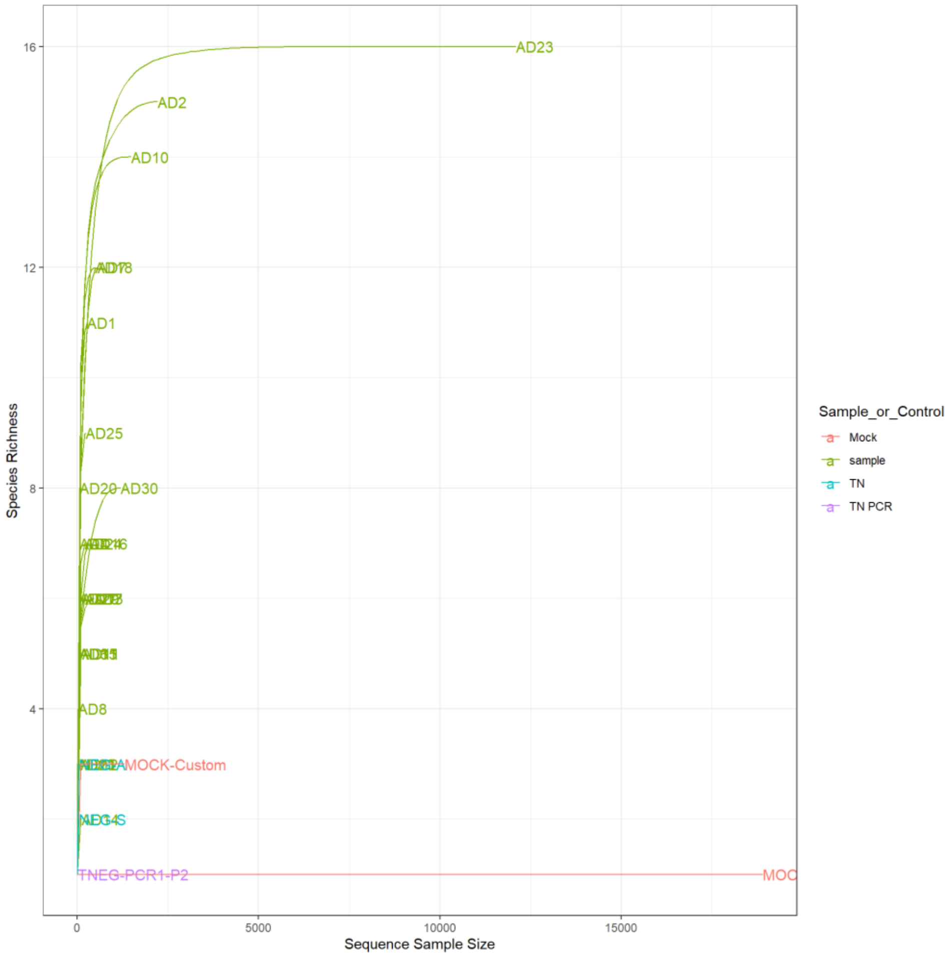

TN: negative control from the extraction step. TN PCR: negative control from the amplification PCR step.

Supplemental Figure 4. Comparison of lung mycobiota between severe AECOPD patients who received antimicrobial therapy within the previous month and those who did not. A. Boxplot of estimated α-diversity by Shannon index. B. Boxplot of estimated α-diversity by Simpson index. C. Boxplot of estimated α-diversity by evenness. D. Non-metric Bray-curtis analysis of β-diversity. Threshold for statistical significance: p=0.05. atb: systemic antibiotics within the previous month.

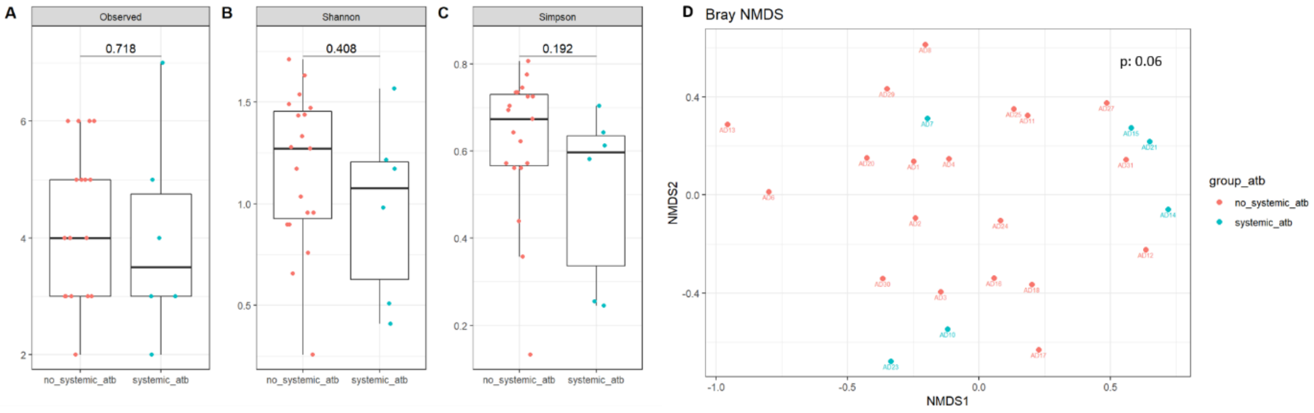

Supplemental Figure 5. Comparison of lung bacteriobiota between severe AECOPD patients who received antimicrobial therapy within the previous month and those who did not. A. Boxplot of estimated α-diversity by Shannon index. B. Boxplot of estimated α-diversity by Simpson index. C. Boxplot of estimated α-diversity by evenness. D. Non-metric Bray-curtis analysis of β-diversity. Threshold for statistical significance: p=0.05. atb: systemic antibiotics within the previous month.

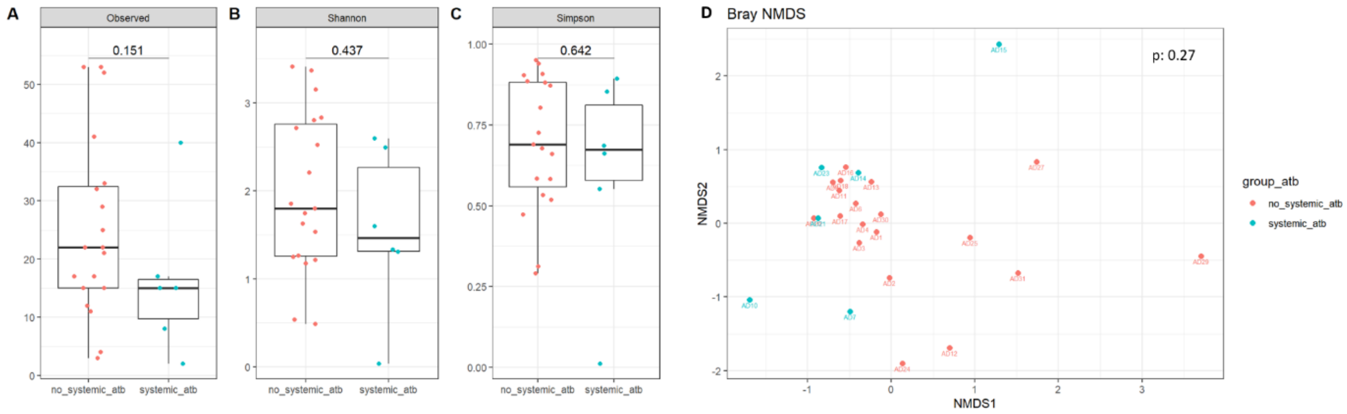

Supplemental Figure 6. Comparison of lung mycobiota between severe AECOPD patients who receive long-term inhaled corticosteroids therapy and those who do not. A. Boxplot of estimated α-diversity by Shannon index. B. Boxplot of estimated α-diversity by Simpson index. C. Boxplot of estimated α-diversity by evenness. D. Non-metric Bray-curtis analysis of β-diversity. Threshold for statistical significance: p=0.05. icortic: long-term inhaled corticosteroids therapy

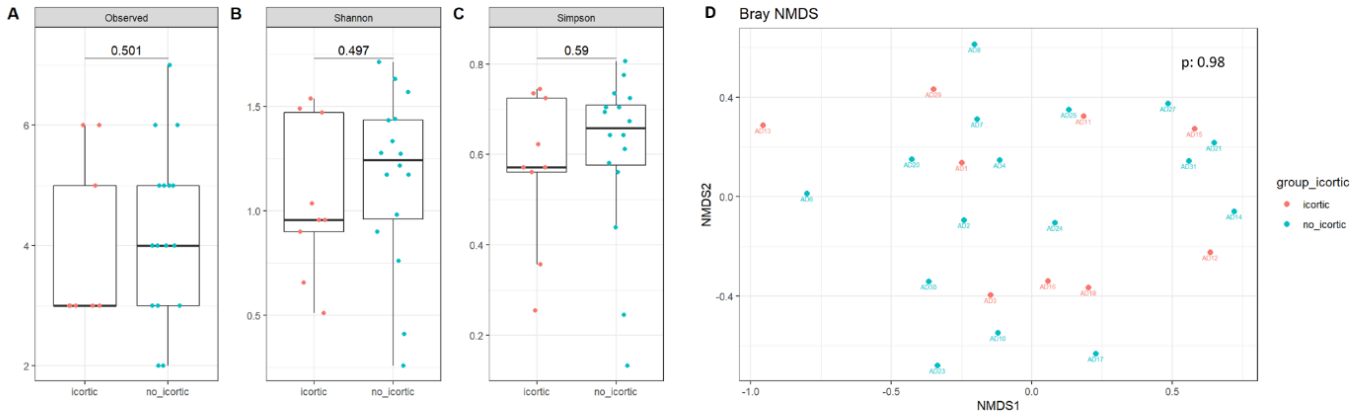

Supplemental Figure 7. Comparison of lung bacteriobiota between severe AECOPD patients who receive long-term inhaled corticosteroids therapy and those who do not. A. Boxplot of estimated α-diversity by Shannon index. B. Boxplot of estimated α-diversity by Simpson index. C. Boxplot of estimated α-diversity by evenness. D. Non-metric Bray-curtis analysis of β-diversity. Threshold for statistical significance: p=0.05. icortic: long-term inhaled corticosteroids therapy

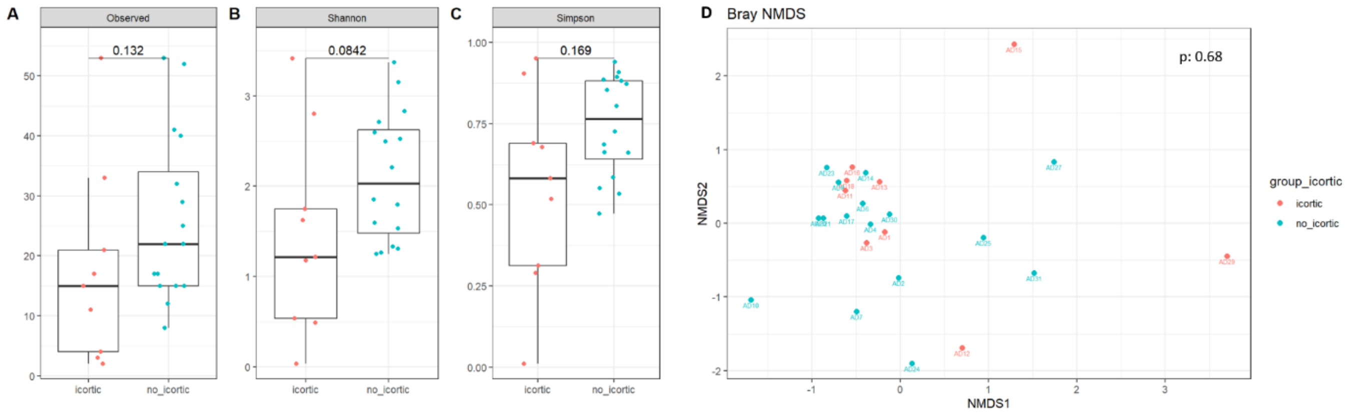

Supplemental Figure 8. Comparison of lung mycobiota between severe AECOPD patients who received systemic corticosteroids therapy within the previous month and those who did not. A. Boxplot of estimated α-diversity by Shannon index. B. Boxplot of estimated α-diversity by Simpson index. C. Boxplot of estimated α-diversity by evenness. D. Non-metric Bray-curtis analysis of β-diversity. Threshold for statistical significance: p=0.05. corticotherapy: corticosteroids therapy within the previous month.

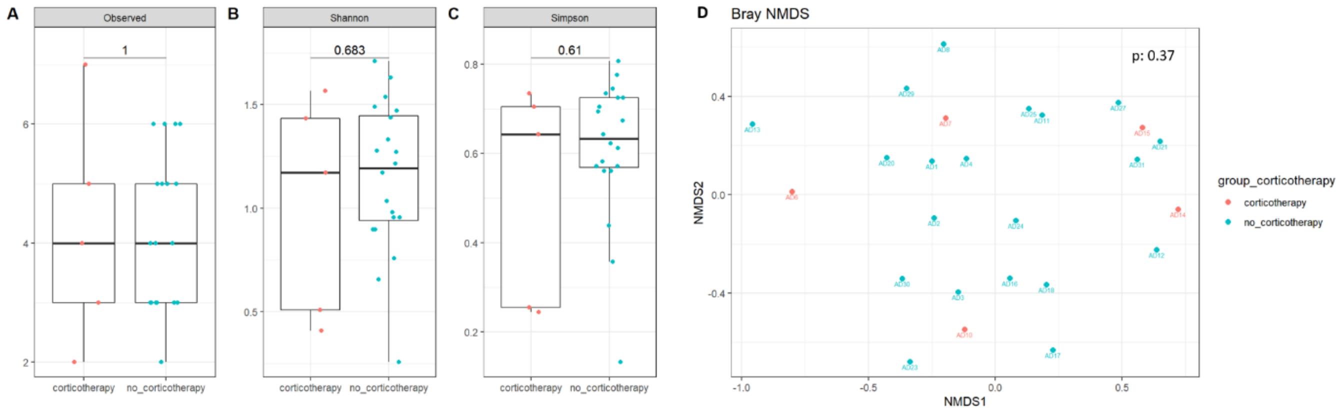

Supplemental Figure 9. Comparison of lung bacteriobiota between severe AECOPD patients who received systemic corticosteroids therapy within the previous month and those who did not. A. Boxplot of estimated α-diversity by Shannon index. B. Boxplot of estimated α-diversity by Simpson index. C. Boxplot of estimated α-diversity by evenness. D. Non-metric Bray-curtis analysis of β-diversity. Threshold for statistical significance: p=0.05. corticotherapy: corticosteroids therapy within the previous month.

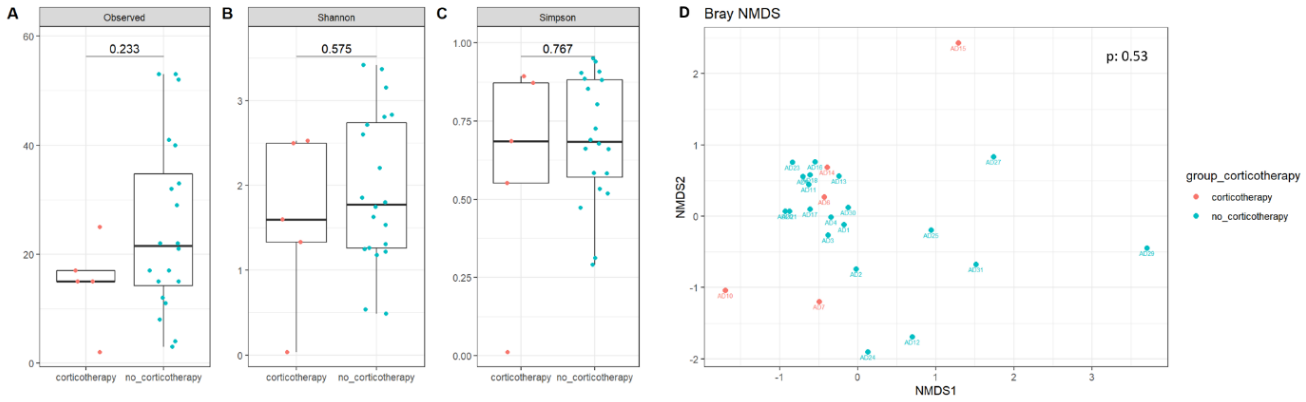

Supplemental Figure 10. Lung mycobiota phylum relative abundance.

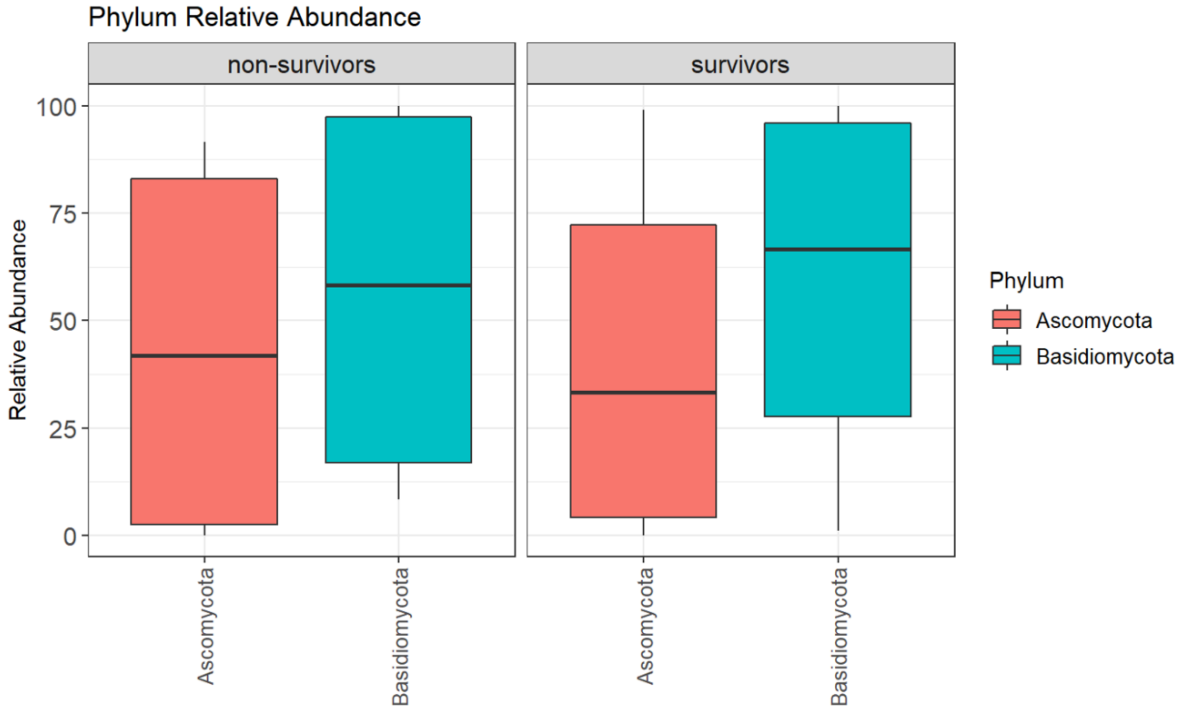

Supplemental Figure 11. Lung bacteriobiota phylum relative abundance.

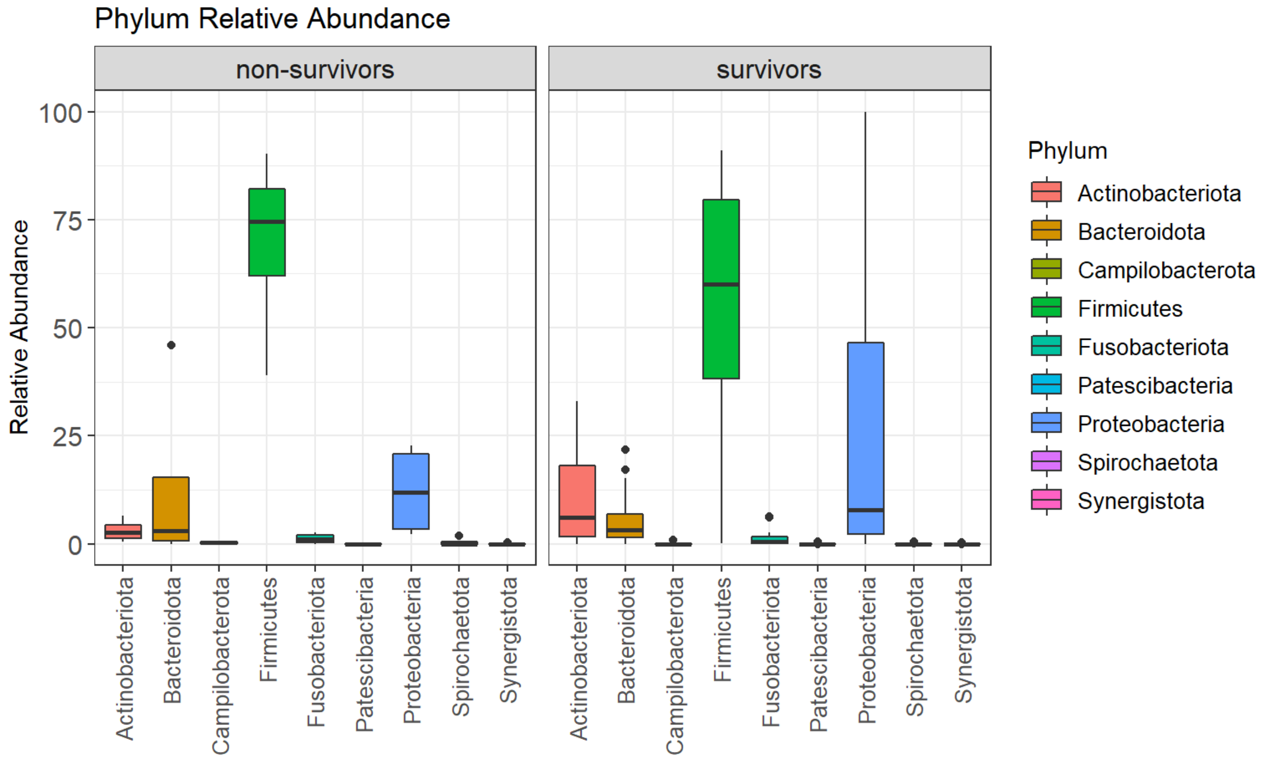

Mock

ZymoBIOMICS® Microbial Community Standard
Listeria monocytogenes - 12%
Pseudomonas aeruginosa - 12%
Bacillus subtilis - 12%
Escherichia coli - 12%
Salmonella enterica - 12%
Lactobacillus fermentum - 12%
Enterococcus faecalis - 12%
Staphylococcus aureus - 12%
Saccharomyces cerevisiae - 2%
Cryptococcus neoformans - 2%

r16SRNA coding gene home-made mock microbial community standards
Streptococcus mitis (400 µL)
Streptococcus oralis (400 µL)
Pseudomonas aeruginosa (200 µL)
Stenotrophomonas maltophilia (200 µL)
Staphylococcus epidermidis (400 µL)
Staphylococcus aureus (400 µL)
Acinetobacter baumannii (400 µL)
Klebsiella pneumoniae (200 µL)
Proteus mirabilis (200 µL)
Serratia marcescens (400 µL)
Lactobacillus spp. (200 µL)
Escherichia coli (ATCC 25922) (200 µL)
Enterobacter cloacae (200µL)
Enterococcus faecalis (ATCC 29212) (400 µL)

ITS2 coding gene home-made mock microbial community standards
Scedosporium apiospermum (*Pseudallescheria boydii*) (200 µL)
Mucor circillenoides (400 µL)
Candida lusitaniae (*Clavispora lusitaniae*) (400 µL)
Scedosporium aurantiacum (400 µL)
Lomentospora prolificans (*Scedosporium prolificans*) (200 µL)
Aspergillus fumigatus (400 µL)
Aspergillus flavus (200 µL)
Aspergillus terreus (200 µL)
Penicillium griseofulvum (200 µL)
Fusarium solani (200 µL)
Candida dubliniensis (400 µL)
Candida albicans (ATCC 5314) (400 µL)
Rhodotorula mucilaginosa (400 µL)
Exophiala dermatitidis (400 µL)

Script for 16S RNA coding gene assignation

```
---
title: "Assignation 16S"
author: "Renaud Prevel & Raphael Enaud"
date: '`r format(Sys.time(), "%d %m %Y")`'

header-includes:
  - \usepackage{color, fancyvrb}
output:
  rmdformats::readthedown:
    highlight: kate
    numbersections : yes
  html_document:
    fig_height: 7
    fig_width: 10
---


```{r knitrinit, include=FALSE, warning=FALSE, message=FALSE, cache=FALSE}

library(rstudioapi)
setwd(dirname(rstudioapi::getActiveDocumentContext()$path))
load("./assignation.rda")

library(knitr)
library(rmdformats)
library("kableExtra")
library(DESeq2)
library("ggplot2")
library("readxl")
library("dplyr")
library(knitr)
library(rmdformats)
library(psy)
library(Rcpp)
library(nnet)
library(dplyr)
library(ggplot2)
library(psy)
library(prettyR)
library(corrplot)
library(readr)
library(questionr)
library(finalfit)
library(labelled)
library("dada2")
library(ShortRead)
library(Biostrings)
library(DECIPHER)
library("plyr")
theme_set(theme_bw())
```
```

```
library (phyloseq)
library(BiocStyle)
library(ape)
library(tidyr)
library(broom)
library(dplyr)
library(Hmisc)
library (microbiome)
library (picante)
library(plyr)
library(reshape2)
library(doBy)
library(RVAideMemoire)
library (vegan)
library(heatmap.plus)
library(RColorBrewer)
library(gplots)
library(kableExtra)
library(gridExtra)
library(knitr)
library(microbiomeSeq)
library(adespatial)
library(ggpubr)
library (devtools)
library(yingtools2)
library("microDecon")

```

````
```{r, include=FALSE}

# Creation of the phyloseq object

samdf<- read_xlsx("./Sample Data.xlsx")
samples_df <- sample_data(samdf)

rownames(samples_df) <- samples_df$sample
samples_df$Groupe <- factor(samples_df$Groupe)
samples_df$Sample_or_Control <- factor(samples_df$Sample_or_Control)

phylo <- phyloseq(otu_table(seqtab.nochim, taxa_are_rows=FALSE),
        sample_data(samples_df),
        tax_table(taxa))

dna <- Biostrings::DNAStringSet(taxa_names(phylo))
names(dna) <- taxa_names(phylo)
phylo <- merge_phyloseq(phylo, dna)
taxa_names(phylo) <- paste0("ASV", seq(ntaxa(phylo)))
````
````

````
phylo
```

# Check the number of reads

```{r, echo=FALSE}
df <- as.data.frame(sample_data(phylo)) # Put sample_data into a ggplot-friendly data.frame
df$LibrarySize <- sample_sums(phylo)
df <- df[order(df$LibrarySize),]
df$Index <- seq(nrow(df))
ggplot(data=df, aes(x=Index, y=LibrarySize, color=Sample_or_Control, label = sample)) + geom_point()

kableExtra::kable(sort(sample_sums(phylo))) %>%
  kable_styling() %>%
  scroll_box(width = "100%", height = "600px")
```

# Selection of bacterial ASVs
```{r, echo=FALSE}
phylo = subset_taxa(phylo, Kingdom=="Bacteria")
phylo

#Removal of phylum with a single taxa
phylo <- subset_taxa(phylo, !is.na(Phylum) & !Phylum %in%
c("","Bdellovibrionota","Chloroflexi","Deinococcota","<NA>","NA"))

phylo
```

# Filtering of minority ASVs

```{r, echo=FALSE}

# ASV present in less than 3 samples
condition <- function(x) { sum(x > 0) >= 2 }
taxaToKeep <- filter_taxa(phylo, condition)
phylo <- prune_taxa(taxaToKeep, phylo)
phylo = prune_samples(names(which(sample_sums(phylo) >= 1)), phylo)
phylo = prune_taxa(names(which(taxa_sums(phylo) >= 1)), phylo)
phylo

```

# Rarefaction curves
```{r, include=FALSE, warning=FALSE, message=FALSE, cache=FALSE, fig.width = 10, fig.height= 10}

library(vegan)
````

library(ggplot2)
library("ranacapa")
phylo = prune_samples(names(which(sample_sums(phylo) >= 1)), phylo)
rarefactioncurves = ggrare(phylo, step = 100, label = "sample", color = "Sample_or_Control",se = FALSE)
```

```{r, echo=FALSE, warning=FALSE, message=FALSE, cache=FALSE, fig.width = 10, fig.height= 10}
rarefactioncurves
```

Beta diversity

Measurement of Beta Diversity by Bray Curits
MDS representation (multi-dimensional scaling)

```{r, include=FALSE, warning=FALSE, message=FALSE, cache=FALSE, fig.width = 10, fig.height= 10}

# Transform data to proportions as appropriate for Bray-Curtis distances
ps.prop <- transform_sample_counts(phylo, function(otu) otu/sum(otu))
ord.nmds.bray <- ordinate(ps.prop, method="MDS", distance="bray")

```
```{r, echo=FALSE, warning=FALSE, message=FALSE, cache=FALSE, fig.width = 10, fig.height= 10}
plot_ordination(ps.prop, ord.nmds.bray, type="samples", label = "sample", color = "Sample_or_Control", title="MDS")

```

Representation NMDS (Non-metric multi-dimensional scaling)
```{r, include=FALSE, warning=FALSE, message=FALSE, cache=FALSE, fig.width = 10, fig.height= 10}
ord.nmds.bray <- ordinate(ps.prop, method="NMDS", distance="bray")

```
```{r, echo=FALSE, warning=FALSE, message=FALSE, cache=FALSE, fig.width = 10, fig.height= 10}
plot_ordination(ps.prop, ord.nmds.bray, type="samples", label = "sample", color = "Sample_or_Control",title="NMDS")

```

Beta diversity tree (with Bray Curtis dissimilarity index)
```{r, include=FALSE, warning=FALSE, message=FALSE, cache=FALSE}
library("ape")
random_tree = rtree(ntaxa(phylo), rooted=TRUE, tip.label=taxa_names(phylo))

phylo1 = merge_phyloseq(phylo,random_tree)
phylo1

~~~~
# Dissimilarity measurement with Bray Cutris
GPUF <- round(distance(phylo1,"bray"),3)
```


```{r, echo=FALSE, warning=FALSE, message=FALSE, cache=FALSE, fig.width = 7, fig.height= 26}

# Creating a tree
## Manually define color-shading vector based on sample type.
colorScale <- rainbow(length(levels(getVariable(phylo1, "Sample_or_Control"))))
cols <- colorScale[getVariable(phylo1, "Sample_or_Control")]
GP.tip.labels <- as(getVariable(phylo1, "sample"), "character")
GP.hclust <- hclust(GPUF, method = "average")

plot(as.phylo(GP.hclust), show.tip.label = TRUE, tip.color = "white")
tiplabels(GP.tip.labels, col = cols, frame = "none",adj = -0.05,cex = 0.7)
```


Removal of negative controls and Mock

```{r, echo=FALSE, warning=FALSE, message=FALSE, cache=FALSE}
phylo <- subset_samples(phylo, Sample_or_Control =="sample")
phylo = prune_taxa(names(which(taxa_sums(phylo) >= 1)), phylo)
phylo

```


Resume of the final dataset


```{r, echo=FALSE, warning=FALSE, message=FALSE, cache=FALSE}
summarize_phyloseq(phylo)
```

saving the global environment

```{r, echo=FALSE, warning=FALSE, message=FALSE, cache=FALSE}
save(phylo, list,file="Phylo pre processed.rda")
```
~~~~

Script for ITS2 coding gene assignation

````
---
title: "ITS Assignment"
author: "Renaud Prevel & Raphael Enaud"
date: '`r format(Sys.time(), "%d %m %Y")`'

header-includes:
  - \usepackage{color, fancyvrb}
output:
  rmdformats::readthedown:
    highlight: kate
    numbersections : yes
  html_document:
    fig_height: 7
    fig_width: 10
---


```{r knitrinit, include=FALSE, warning=FALSE, message=FALSE, cache=TRUE}

# In "path", create a folder "Fastq" with the sequences R1 and R2.


library(knitr)
library(rmdformats)
library("kableExtra")
library(DESeq2)
library("ggplot2")
library("readxl")
library("dplyr")
library(knitr)
library(rmdformats)
library("kableExtra")
library(DESeq2)
library(psy)
library(Rcpp)
library(nnet)
library(dplyr)
library(ggplot2)
library(psy)
library(prettyR)
library(corrplot)
library(readr)
library(questionr)
library(finalfit)
library(labelled)
library("dada2")
library(ShortRead)
library(Biostrings)
library(DECIPHER)
library("plyr")
````
````

````
library("phyloseq")
library("ranacapa")
theme_set(theme_bw())

library(rstudioapi)
setwd(dirname(rstudioapi::getActiveDocumentContext()$path))
path <- file.path(".")
input <-list.dirs(path, full.names = F, recursive = F)

qual <-"./quality/"
outp <-"./output/"
fastq <- "./Fastq"
R1 <- "./R1"

fnFs <- sort(list.files(fastq, pattern = "_R1_001.fastq.gz", full.names = TRUE))
fnRs <- sort(list.files(fastq, pattern = "_R2_001.fastq.gz", full.names = TRUE))

ITS2 Primers (ITS7F / 3271R):
FWD <- "GTGARTCATCGAATCTTT"
REV <- "GATATGCTTAAGTTCAGCGGGT"
```

```{r, include=FALSE, warning=FALSE, message=FALSE, cache=FALSE}

# Checking the presence and orientation of primers

allOrients <- function(primer) {
  # Create all orientations of the input sequence
  require(Biostrings)
  dna <- DNAString(primer)  # The Biostrings works w/ DNAString objects rather than character vectors
  orients <- c(Forward = dna, Complement = Biostrings::complement(dna), Reverse = reverse(dna),
               RevComp = reverseComplement(dna))
  return(sapply(orients, toString))  # Convert back to character vector
}
FWD.orients <- allOrients(FWD)
REV.orients <- allOrients(REV)
FWD.orients
REV.orients

# Pre filtration
fnFs.filtN <- file.path(fastq, "filtN", basename(fnFs)) # Put N-filterd files in filtN/ subdirectory
fnRs.filtN <- file.path(fastq, "filtN", basename(fnRs))
out <- filterAndTrim(fnFs, fnFs.filtN, fnRs, fnRs.filtN, maxN = 0, multithread = TRUE)

primerHits <- function(primer, fn) {
  # Counts number of reads in which the primer is found
  nhits <- vcountPattern(primer, sread(readFastq(fn)), fixed = FALSE)
  return(sum(nhits > 0))
}
````

```

Number of primers found :

```{r, echo=FALSE, warning=FALSE, message=FALSE, cache=FALSE}
rbind(FWD.ForwardReads = sapply(FWD.orients, primerHits, fn = fnFs.filtN[[1]]),
      FWD.ReverseReads = sapply(FWD.orients, primerHits, fn = fnRs.filtN[[1]]),
      REV.ForwardReads = sapply(REV.orients, primerHits, fn = fnFs.filtN[[1]]),
      REV.ReverseReads = sapply(REV.orients, primerHits, fn = fnRs.filtN[[1]]))
```

Primer removal

```{r, include=FALSE, warning=FALSE, message=FALSE, cache=FALSE}

cutadapt <-
"C:/Users/EnaudR/AppData/Local/Packages/PythonSoftwareFoundation.Python.3.9_qbz5n2kfra8p0
/LocalCache/local-packages/Python39/Scripts/cutadapt.exe" # CHANGE ME to location on your
machine


R1 <- file.path(path, "R1")
if(!dir.exists(R1)) dir.create(R1)
R2 <- file.path(path, "R2")
if(!dir.exists(R2)) dir.create(R2)

fnFs.cut <- file.path(R1, basename(fnFs))
fnRs.cut <- file.path(R2, basename(fnRs))

FWD.RC <- dada2:::rc(FWD)
REV.RC <- dada2:::rc(REV)
# Trim FWD and the reverse-complement of REV off of R1 (forward reads)
R1.flags <- paste("-g", FWD, "-a", REV.RC)
# Trim REV and the reverse-complement of FWD off of R2 (reverse reads)
R2.flags <- paste("-G", REV, "-A", FWD.RC)

# Run Cutadapt
for(i in seq_along(fnFs)) {
  system2(cutadapt, args = c(R1.flags, R2.flags, "-n", 2, # -n 2 required to remove FWD and REV from
reads
                             "-m", 20, "-o", fnFs.cut[i], "-p", fnRs.cut[i], # output files, filtrage des reads au
minimum 20 pb
                             fnFs.filtN[i], fnRs.filtN[i])) # input files
}
```

```{r, echo=FALSE, warning=FALSE, message=FALSE, cache=FALSE}
rbind(FWD.ForwardReads = sapply(FWD.orients, primerHits, fn = fnFs.cut[[1]]),
      FWD.ReverseReads = sapply(FWD.orients, primerHits, fn = fnRs.cut[[1]]),
      REV.ForwardReads = sapply(REV.orients, primerHits, fn = fnFs.cut[[1]]),
      REV.ReverseReads = sapply(REV.orients, primerHits, fn = fnRs.cut[[1]]))

````
```

```{r, include=FALSE, warning=FALSE, message=FALSE, cache=FALSE}

path <- file.path(".")
input<-list.dirs(path, full.names = F, recursive = F)

qual<-"./quality/"
outp<-"./output/"
R1 = "./R1"

write.fasta.dada<-function(dada2, file){
  seqs<-dada2::getSequences(dada2)
  hash<-paste0(">",sapply(seqs, openssl::sha1, USE.NAMES = F))
  write(c(rbind(hash, seqs)),file)
}

for (R1 in R1){
  # List FASTQ files
  fq <-list.files(file.path(path,"R1"), pattern="_001.fastq.gz")
    # Extract sample names, assuming filenames have format: SAMPLENAME_XXX.fastq
  sample.names <- sapply(strsplit(fq, "_"), `[`, 1)

  # Add the full path
  fq <- file.path(R1, fq)

  filt_path <- file.path(qual,"R1") # Place filtered files in subdirectory
  dir.create(filt_path, recursive = T)
  # Prepare future filenames
  filt <- file.path(filt_path, paste0(sample.names, ".filt.fastq.gz"))


  out.ee <- ldply(1:length(sample.names),function(i) {
    fastqFilter(fn = fq[i],
              fout = filt[i],
              trimLeft = 0,minLen = 100,
              maxN=0, maxEE = 1, rm.phix=TRUE,
              compress=TRUE)
  })

  # Dereplication
  derep <- llply(1:length(sample.names), function(i){
    derepFastq(filt[i], verbose=TRUE)
  })
  names(derep)<-sample.names

  # Learn errors
  err <- learnErrors(derep, multithread=3)


  # Get variants
````

````
  dada_obj<-dada(derep, err=err, multithread = 3)
  # Create output directory
  #outp_rds<-file.path(outp,R1, "RDS")
  #dir.create(outp_rds, recursive = T)
  #saveRDS(dada_obj, file = paste0(outp_rds,"/",R1,".RDS"), compress = "gzip")

  # Produce Variant Table
  seqtab<-makeSequenceTable(dada_obj)



  # Remove Chimeras
  seqtab.nochim <- removeBimeraDenovo(seqtab, verbose=TRUE)

}
```



# List of samples

```{r, echo=FALSE, warning=FALSE, message=FALSE, cache=FALSE}
sample.names
```


# Checking reading quality profiles



```{r, echo=FALSE, warning=FALSE, message=FALSE, cache=FALSE}
plotQualityProfile(fq[1:2])
```

# Filtering reads

```{r, echo=FALSE, warning=FALSE, message=FALSE, cache=FALSE}
# Place filtered files in filtered/ subdirectory

```



# Learning about error rates


```{r, echo=FALSE, warning=FALSE, message=FALSE, cache=FALSE}
plotErrors(err, nominalQ=TRUE)
```



# Bulding an ASVs table
```{r, echo=FALSE, warning=FALSE, message=FALSE, cache=FALSE}
````

seqtab <- makeSequenceTable(dada_obj)
dim(seqtab)
```

Checking the distribution of sequence lengths
```{r, echo=FALSE, warning=FALSE, message=FALSE, cache=FALSE}
options(max.print="500")
table(nchar(getSequences(seqtab)))


```

Remove chimeras
```{r, echo=FALSE, warning=FALSE, message=FALSE, cache=FALSE}
seqtab.nochim <- removeBimeraDenovo(seqtab, verbose=TRUE)
dim(seqtab.nochim)
sum(seqtab.nochim)/sum(seqtab)
table(nchar(getSequences(seqtab.nochim)))
```


Summary of the different steps of the pipeline

```{r, include=FALSE, warning=FALSE, message=FALSE, cache=FALSE}
getN <- function(x) sum(getUniques(x))
track <- cbind(out, sapply(dada_obj, getN), rowSums(seqtab.nochim))

## If processing a single sample, remove the sapply calls: e.g. replace sapply(dadaFs, getN) with getN(dadaFs)
colnames(track) <- c("input", "filtered", "denoised", "nonchim")
rownames(track) <- sample.names
```

```{r, echo=FALSE, warning=FALSE, message=FALSE, cache=FALSE}
options(max.print="600")
track

```

Assignment


```{r, include=FALSE, warning=FALSE, message=FALSE, cache=FALSE}
library(stringr)
unite.ref <- "./sh_general_release_dynamic_s_10.05.2021.fasta"  # CHANGE ME to location on your machine
taxa <- assignTaxonomy(seqtab.nochim, unite.ref, multithread = TRUE, tryRC = TRUE)

str_replace_all(taxa, pattern = "k__", replacement = "")
str_replace_all(taxa, pattern = "p__", replacement = "")
str_replace_all(taxa, pattern = "c__", replacement = "")
str_replace_all(taxa, pattern = "o__", replacement = "")

str_replace_all(taxa, pattern = "f__", replacement = "")
str_replace_all(taxa, pattern = "g__", replacement = "")
str_replace_all(taxa, pattern = "s__", replacement = "")

```

Viewing the found assignments :

```{r, echo=FALSE, warning=FALSE, message=FALSE, cache=FALSE}
taxa.print <- taxa  # Removing sequence rownames for display only
rownames(taxa.print) <- NULL
head(taxa.print)
```

# saving the global environment
save(out.ee, fq, seqtab, seqtab.nochim, err, track,sample.names, taxa , file = "./assignation Unite.rda")
...
```
